# Prediction of Mobility Limitations after Hospitalization in Older Medical Patients by Simple Measures of Physical Performance Obtained at Admission to the Emergency Department

**DOI:** 10.1371/journal.pone.0154350

**Published:** 2016-05-19

**Authors:** Ann Christine Bodilsen, Henrik Hedegaard Klausen, Janne Petersen, Nina Beyer, Ove Andersen, Lillian Mørch Jørgensen, Helle Gybel Juul-Larsen, Thomas Bandholm

**Affiliations:** 1 Optimized Senior Patient Program, Clinical Research Centre, Copenhagen University Hospital, Hvidovre, Denmark; 2 Physical Medicine & Rehabilitation Research-Copenhagen (PMR-C); Department of Physical and Occupational Therapy, Copenhagen University Hospital, Hvidovre, Denmark; 3 Section of Biostatistics, Department of Public Health, University of Copenhagen, Copenhagen, Denmark; 4 Musculoskeletal Rehabilitation Research Unit, Department of Physical Therapy, Bispebjerg & Frederiksberg Hospital, University of Copenhagen, Copenhagen, Denmark; 5 The Emergency Department, Copenhagen University Hospital, Hvidovre, Denmark; 6 Department of Orthopaedic Surgery, Copenhagen University Hospital, Hvidovre, Denmark; University of Zaragoza, SPAIN

## Abstract

**Objective:**

Mobility limitations relate to dependency in older adults. Identification of older patients with mobility limitations after hospital discharge may help stratify treatment and could potentially counteract dependency seen in older adults after hospitalization. We investigated the ability of four physical performance measures administered at hospital admission to identify older medical patients who manifest mobility limitations 30 days after discharge.

**Design:**

Prospective cohort study of patients (≥65 years) admitted to the emergency department for acute medical illness. During the first 24 hours, we assessed: handgrip strength, 4-meter gait speed, the ability to rise from a chair (chair-stand), and the Cumulated Ambulation Score. The mobility level 30 days after discharge was evaluated using the de Morton Mobility Index.

**Results:**

A total of 369 patients (77.9 years, 62% women) were included. Of those, 128 (40%) patients had mobility limitations at follow-up. Univariate analyzes showed that each of the physical performance measures was strongly associated with mobility limitations at follow-up (handgrip strength_(women)_, OR 0.86 (0.81–0.91), handgrip strength_(men)_, OR 0.90 (0.86–0.95), gait speed, OR 0.35 (0.26–0.46), chair-stand, OR 0.04 (0.02–0.08) and Cumulated Ambulation Score OR 0.49 (0.38–0.64). Adjustment for potential confounders did not change the results and the associations were not modified by any of the covariates: age, gender, cognitive status, the severity of the acute medical illness, and the Charlson Comorbidity Index. Based on prespecified cut-offs the prognostic accuracy of the four measures for mobility limitation at follow-up was calculated. The sensitivity and specificity were: handgrip strength_(women)_, 56.8 (45.8–67.3), 75.7 (66.8–83.2), handgrip strength_(men)_, 50.0 (33.8–66.2), 80.8 (69.9–89.1), gait speed, 68.4 (58.2–77.4), 81.4 (75.0–86.8), chair-stand 67.8 (58.6–76.1), 91.8 (86.8–95.3), and Cumulated Ambulation Score, 40.2 (31.6–49.2), 92.0 (87.1–95.4), respectively.

**Conclusion:**

Physical performance measures, particularly chair-stand and gait speed assessed at admission to an emergency department, were able to identify mobility limitation in acutely admitted older medical patients 30 days after hospital discharge.

## Introduction

Functional status—particularly mobility—is an important manifestation of illness in older adults, and a relevant prognostic factor of adverse health events in geriatric treatment across diagnoses [1–4]. According to the National Health Interview Surveys from 2005 limited mobility affected roughly 40% of older adults (≥65 years) in Denmark [5]. Mobility limitations are associated with functional decline, nursing home placements, and increased mortality rates in both community-living and hospitalized older adults [6–14]. Indeed, mobility limitation is an early sign of functional decline in older adults and coexists with reduced muscle strength in upper and lower extremities [1,15–19]. Mobility limitations following medical hospitalization are often sustained [20], and studies found that only 30% of patients with functional decline during medical hospitalization regained their loss within one year after discharge [21,22]. These findings indicate that the recovery of functional loss is compromised in many older medical patients, which results in a persistent lower functional level than before the hospitalization [20,22,23], often initiating a vicious circle with restricted social activities, reduced physical activity, and further dependence [1,20]. On the other hand, Boyd and coworkers found that older medical patients who regained their pre-hospitalization function within 30 days after discharge compared to patients who did not, had lower mortality and maintained their functional level one year after discharge [22]. Functional decline could potentially be reduced if relevant interventions were offered to medical patients with a high risk of persistent mobility limitations after hospitalization. However, this requires early identification of at-risk patients.

The current tools to identify older patients with an increased risk of functional decline following acute illness and hospitalization—like the Identification of Seniors at Risk and Hospital Admission Risk Profile [24,25] are actually poor at predicting functional decline [24,25]. Physical performance measures such as the Short Physical Performance Battery (SPPB), gait speed (GS), chair stand (CS), and handgrip strength (HGS), have been suggested as “vital signs” of decreased physiological reserve capacity [10,11,26–31], and poor performance in the same measures is associated with an increased risk of current and future mobility limitations [9,10,13,15,17,18,31] in healthy older persons. Notably, performance in GS is nearly as good a predictor of mobility limitations as the full and more time-consuming SPPB [10,13]. Although, poor performance in GS [26,32], SPPB [6–8,33] and HGS [34] is associated with present and future ADL disability in older medical patients, very little is known about the predictive ability of physical performance measures to identify mobility limitations post discharge in older medical patients [8]. Furthermore, existing studies have evaluated physical performance measures close to discharge [6–8], which is too late for planning in-hospital treatment. For this purpose, it is essential that the assessments are carried out at hospital admission.

Accordingly, the objectives of this study were: to determine how four simple physical performance measures administered at hospital admission are associated with mobility limitations 30 days after discharge. Further to investigate the ability of the four measures to predict older medical patients who manifest mobility limitations 30 days after discharge. The assessments were performed at the hospital´s emergency department (ED), and comprised HGS, GS and CS as mentioned above as well as the Cumulated Ambulation Score (CAS). We hypothesized that one or more of the physical performance measures would be able to predict mobility limitations 30 days after discharge in older medical patients.

## Methods

### Ethical Statement

The study was conducted in accordance with the Declaration of Helsinki. Signed informed consent was obtained from all participants. The study was approved by The Danish Data Protection Agency (01596 HVH-2012-005), and the Research Ethics Committees for The Capital Region (H-1-2011-167). When the study was designed, endorsement of registration of observational trials was not as prevalent as today, which is why the trial was not registered.

### Setting

This study was performed in the medical section of the 30-bed ED at Copenhagen University Hospital, Hvidovre, Denmark. In Denmark, the majority of older medical patients (≥65 years) are admitted through the ED [35]. All physicians in the ED at Hvidovre Hospital have the possibility to refer any patient to a geriatric multidisciplinary team and to physical rehabilitation if needed. Furthermore, the geriatric multidisciplinary team selects older patients who need a comprehensive geriatric assessment in a non-systematic way based upon clinical judgement.

### Design and participants

This study is a prospective cohort study, enrolling patients at the admission to the ED from July 2012 to September 2013, and with a follow-up visit at the patients´ home 30 days after discharge. The inclusion criteria were: age ≥65 years and admitted for an acute medical illness. The exclusion criteria were: inability to cooperate, short length of stay that excluded assessment before discharge, inability to understand Danish, admission to an intensive care unit, cancer, terminal disease or patients in isolation. Patients were randomly selected, based on their social security number by the use of a computer-generated list, as more patients met the inclusion criteria than could be included with respect to assessment resources. The reporting of the study follows the Strengthening the Reporting of Observational Studies in Epidemiology (STROBE) guidelines (www.equator-network.org), and selected elements from the Transparent Reporting of a Multivariable Prediction Model for Individual Prognosis or Diagnosis: the TRIPOD Statement (www.equator-network.org), as the study used a single dataset (no validation) to develop a non-multivariable prediction of mobility status 30 days after discharge.

### Outcome variable: mobility

The primary outcome was the mobility level 30 days after discharge assessed with the Danish version of the de Morton Mobility Index (DEMMI) [36]. The DEMMI was assessed based on observation of the patient´s performance in the ED at admission (data not reported) and at the patient´s home 30 days after discharge, respectively. DEMMI is an interval-level unidimensional mobility measure that was constructed using Rasch analysis and originally developed for objective assessment of mobility in older acute medical patients. It consists of 15 hierarchical-items that assess mobility across a spectrum from bed-bound to independent mobility [36]. DEMMI is reliable and valid in older persons across different health-care settings, i.e. in the community, and during sub-acute and acute hospitalization [37–40]. DEMMI scores range from 0 to 100 with lower scores indicating mobility limitations [36]. The mobility level at follow-up was dichotomized as “Limited mobility” (<62 points) versus “high mobility” (≥62 points), according to normative values from community-living older adults [41].

### Predictor variables: physical performance measures

The physical performance measures were administered in a pre-defined order to meet the needs of bedridden patients and to reduce fatigue. The order was HGS, GS, CS, and CAS. The four measures were selected based on their usability so they; could be used no matter the functional level of the patient (from no walking ability to independent walking ability), were fast and easy to administer for health-care professionals having a strong focus on securing vacant beds for incoming patients (flow culture) [42]. Furthermore, the measures required modest space and equipment, making them realistically implementable in the ED. The feasibility and inter-tester reliability of the measures—when administered in this hospital setting and in this patient population—have been reported previously [43].

Assessment of physical performance was performed at admission to the ED according to that described previously [43]. Briefly, HGS was assessed in the dominant hand using a hand-grip dynamometer (Saehan, Digi-II). Patients were tested in a sitting position with their elbow in 90° of flexion. The grip width was adjusted according to the size of the hand; for the majority of patients the dynamometer handle was in the second position. Patients were instructed to squeeze the handle as forcefully as possible. After one practice trial three valid trials were recorded. If the third trial elicited the highest value additional trials were performed. The highest value in kilograms was used as the data point. GS was assessed over a 4-m course and the patient was allowed to use a walking aid, if needed. Patients walked at their usual pace from a static start. To reduce the effect of deceleration, patients were instructed to walk towards a visual goal and were stopped after walking 5.5 m. The timer was started upon the first foot-step and stopped when the first foot crossed the 4-m end line. The faster of two trials was used as the data point [43].

Based on our feasibility study [43], a repeated CS test was not chosen, as almost half of the included older medical patients in the feasibility study were unable to rise from a chair without using the armrests. Therefore, CS was evaluated as either: able to rise from a chair with arms folded across the chest (CS_+_); or unable to rise from a chair with arms folded across the chest (CS_−_). Dependency in basic mobility was evaluated using the CAS [44,45]. The CAS quantifies the patient’s dependence in performing three activities: getting into and out of bed, sit-to-stand from a chair, and walking. Each activity is scored on an ordinal scale from 0 to 2 (0 = not able to, 1 = with guidance/support, 2 = independently). The resulting total score ranges from 0 (no basic mobility) to 6 (independent basic mobility). CAS was scored using observations based on the physical performance measures presented above. The CAS is feasible in hospitalized older patients [43,45], and it is a valid predictor of length of hospitalization, discharge to own home, 30-day mortality, and postoperative medical complications in older patients with hip fracture [44].

#### Cut-off values for physical performance measures

For HGS and GS, prognostic accuracy was quantified according to cut-offs from the literature used to identify older community-living adults at risk of various adverse health events, including functional decline [11,27], mobility limitations [13,15,17,18,46] and death [11,13,27,47]. For HGS, different cut-offs as markers for mobility limitations exist; for women: 16 kg, 20 kg, and 21 kg, and for men: 26 kg, 30 kg, and 37 kg [15,17,46]. Our analysis was based on reported cut-offs from The Foundation for the National Institutes of Health Sarcopenia Project [17,18]. Accordingly, poor performance for HGS was ≤ 16 kg for women (HGS_W_) and ≤ 26 kg for men (HGS_M_) [17,18]. Correspondingly, several cut-offs for adverse health outcomes, including mobility limitations, exist for GS: 0.6 m/s 0.8 m/s and 1.0 m/s [11,13,27], and we chose a cut-off for poor performance for gait speed of ≤ 0.6 m/s, based on recommendations for frail older adults [11,27]. Regarding CS, poor performance was defined as; unable to rise from a chair with arms folded across the chest (CS-). For CAS, poor performance was quantified according to cut-offs discriminating full independence from some degree of dependency in basic mobility. Accordingly, ≤ 5 points indicated some dependency in basic mobility.

### Descriptive data

Descriptive data were collected using a questionnaire-based interview at admission and at follow-up, and included: cognitive status, limitations in walking and ADL. Cognitive status was assessed with the Short Orientation-Memory-Concentration Test. The score ranges from 0 to 28 points, with lower scores indicating poorer cognitive ability [48].

Self-reported premorbid functional independence relating to indoor and outdoor walking was assessed with the New Mobility Score (based on the patient’s recall of independence in walking two weeks before the current admission) and on the day of admission [49,50]. The score ranges from 0 to 9 points, with lower scores reflecting dependency. ADL at admission and follow-up was assessed with the Barthel Index 20 [51]. The score ranges from 0 to 20 points, with lower scores representing dependency. Data concerning physical rehabilitation in the time period from discharge to the follow-up visit were retrospectively collected by interview. Comorbidities and causes of admission were extracted from the National Patient Registry based on the International Classification of Diseases, 10th Edition (ICD-10). To describe the impact of comorbidities, the Charlson Comorbidity Index [52] was calculated based on a 10-year prevalence of the ICD-10 codes registered prior to the index admission [53]. Cause of admission was grouped according to ICD-10 chapters.

### Vital signs

To describe the severity of the acute medical illness, the Early-Warning-Score was assessed prior to the physical assessments both at admission and at follow-up [54]. The Early-Warning-Score is based on seven vital signs: level of consciousness, temperature, heart rate, arterial blood pressure, respiratory rate, peripheral oxygen saturation, and oxygen therapy [54] and scored on an ordinal scale from 0 to 20, with higher scores reflecting severe illness and higher mortality risk.

### Outcome assessors

Trained health-care professionals with different educational background performed the physical assessments and the questionnaire-based interview of all patients within the first 24-hours after admission. DEMMI was reassessed and a questionnaire-based interview was performed at the follow-up visit 30 days after discharge. A core team consisting of a physiotherapist, a medical student and a medical doctor performed 80% of all the assessments with a back-up team of four investigators i.e. a nurse, two physiotherapists and a research assistant performing the last 20% of the assessments. The same investigator, made all assessments in the same patient. This was done within the limits of logistics.

### Sample size

This study was performed as a part of the Disability in older medical patients (DISABLMENT) cohort aimed to investigate the ability of physical performance measures and biomarkers to predict adverse health events following acute medical hospitalization one year after discharge. The sample size was calculated based on a Chi-square test for two proportions using a power of 80% and a p-value of 0.05. The proportion of patients with poor performance in each of the physical performance measure was assumed to be 50%, with the assumptions that the proportions with limited mobility 30 days after hospitalization were assumed to be 0.5 in patients with low physical performance, whereas only 0.3 of the patients with high performance at baseline had limited mobility 30 days after discharge. The sample size calculation indicated that 94 subjects were needed in each group. To account for an expected drop-out rate of 50% during the first year, a total sample of 376 patients was included.

## Statistical analyzes

The study sample and patients who declined to participate were compared with respect to categorical variables using the Chi-squared test, and Kruskal-Wallis test for ordinal or continuous variables. Baseline characteristics with medians and interquartile range (IQR) are shown for continuous variables, and absolute and relative frequencies are shown for categorical variables. Potential differences in baseline variables between patients with limited mobility versus high mobility were evaluated with the Kruskal-Wallis test for continuous variables and the Chi-square test for categorical variables.

For each of the physical performance measures, odds ratios (OR) and 95% confidence interval (95% CI) for limited mobility at follow-up were initially calculated using logistic regression analysis. A R-squared value (R^2^) was calculated for limited mobility based on the methods described by Tjur [55] for each of the physical performance measures. The analysis for HGS was stratified according to gender. The explanatory variables were HGS_Men_ (HGS_M_), HGS_Women_ (HGS_W_), GS, CS, and CAS, and the response variable was limited mobility at follow-up. Furthermore, the models were adjusted for potential preselected confounders: age, gender, cognitive status, the severity of the acute medical illness based on the Early-Warning-Score, and the impact of comorbidities based on Charlson Comorbidity Index categorized in three groups with 0, 1–2 and 3 or more comorbidities. Age, cognitive status, and the Early-Warning-Score were entered as continuous covariates. Moreover, to test for possible modifiers, the models were extended with interactions between performance measures and the possible confounders. To account for multiple testing, the significance level for the potential confounders and modifiers was p < 0.01. Tests for goodness of fit were performed with the Hosmer-Lemeshow test. Linearity was evaluated by modeling the physical performance measures as a second degree polynomial.

Receiver operating characteristic (ROC) curves were computed to determine the predictive ability of HGS, and GS with respect to limited mobility. An area under the curve > = 0.7 was used as the threshold for adequate predictive accuracy [56]. The preselected cut-offs were then used to calculate the sensitivity, specificity, positive predictive value, negative predictive value, positive likelihood ratio and negative likelihood ratio for the physical performance measures in relation to mobility status. To validate the preselected cut-offs, the optimal cut-off values were estimated for handgrip strength and gait speed, using the Youden Index based on the fitted logistic regressions analysis [56]. Similarly, the Youden Index was used to calculate the maximum sensitivity and specificity for the complete algorithm, including HGS, GS_NW_, CS, CAS and an interaction between HGS and gender to account for gender differences in cut-off values.

The level of significance was set at 0.05, and all statistical tests were two-tailed. The statistical analyzes were performed using Enterprise Guide 6.1 (SAS Institute, Cary, NC, USA).

## Results

### Attendance and dropout

**Fig 1:**
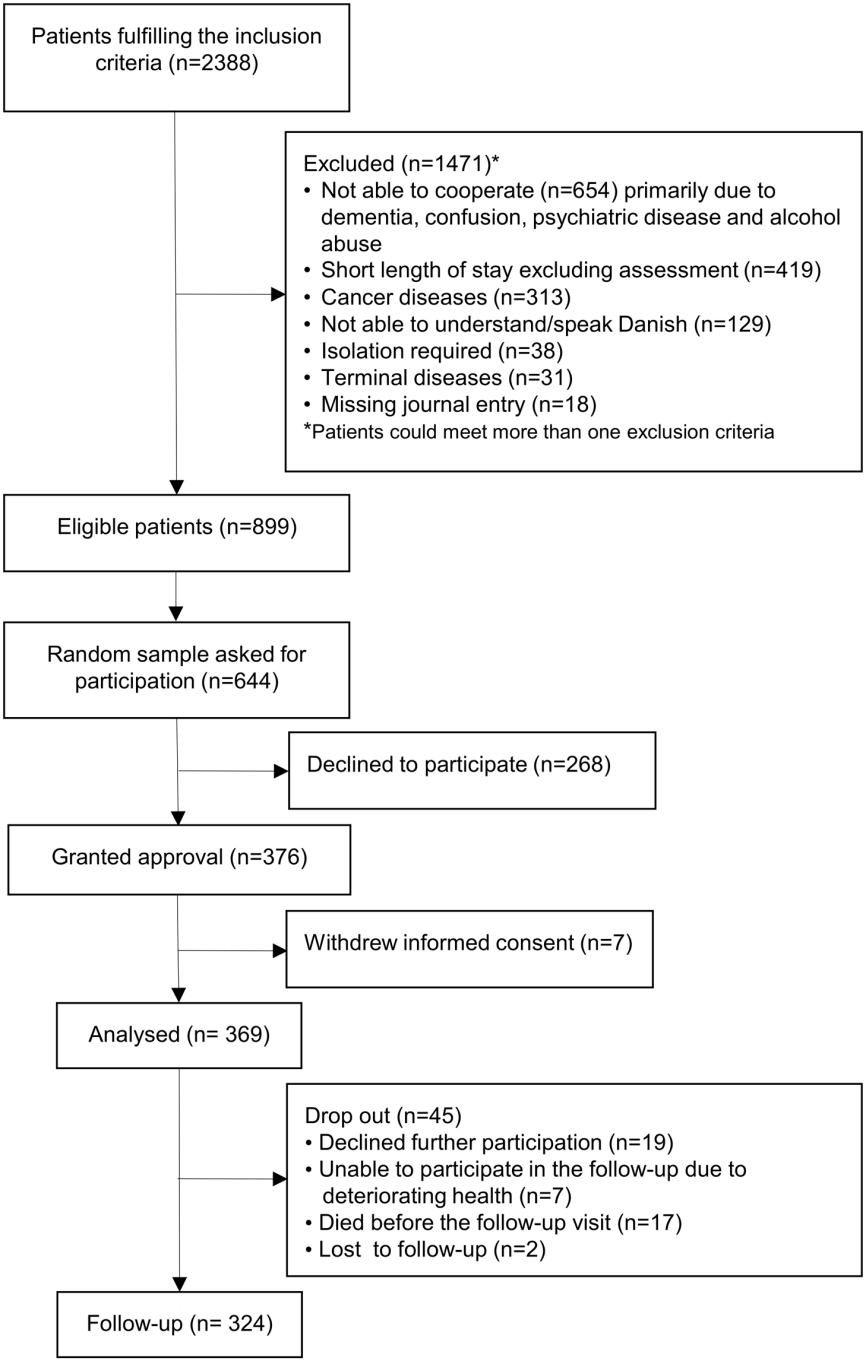
Inclusion of patients in the study (N = 369).

On inclusion days, 2388 medical patients (≥65 years) were admitted to the ED. Of these 1471 were excluded (Fig 1). Of the 899 eligible medical patients, 644 patients were asked to participate and 376 granted approval to participate. Of these, 7 patients withdrew consent before testing. The patients who declined to participate in the study (n = 268) did not differ significantly from the participants (n = 376) regarding age (79.6 versus 78.0 years, p = 0.07) and sex (women: 66 versus 62%, p = 0.32). After patient recruitment was started, we decided to collect data on the status of walking limitations in patients who declined to participate. Thus, 183 of the 268 decliners provided information of mobility limitations (quantified with New Mobility Score). The patients who declined participation had a significantly lower New Mobility Score compared to the study sample both at admission (3 versus 4 points, p = 0.03) and 14 days before admission (6 versus 7 points, p = 0.02). Among the 369 patients who were assessed at admission, 324 patients participated in the follow-up visit. The reasons for not participating in the follow-up visit are shown in Fig 1. Of the 324 patients, 23 patients had DEMMI assessments with missing values in one to three items. In 15 of the 23 patients, the missing values did not affect the mobility status, and the patients were included in analyzes.

### Characteristics of the study sample

Baseline characteristics of the study sample are shown in Table 1. Up to 52 patients had values missing for one or more predictors (Table 1). The main reason for missing values was physical constraints related to the illness preventing assessment and for GS specifically, 23 patients were wheelchair users. Up to three patients declined to participate in test of GS, CS and CAS. At the follow-up, 128 patients had limited mobility and 188 had high mobility. Patients with limited mobility at follow-up had poorer results at admission compared to patients with high mobility (Table 1), regarding all physical performance measures, cognitive status, ADL, and more patients used a walking aid. In addition, they were older, reported more falls, more assistance with self-care and household activities compared to patients with high mobility (Table 1). Further, they had significantly poorer (p<0.001) self-reported mobility (New Mobility Score median = 4) at follow-up compared to those with high mobility (New Mobility Score median = 9). In the time between discharge and the follow-up, 46 patients received physical rehabilitation for more than one session per week. The distribution of rehabilitation was independent of the level of the physical performance measures at baseline, except for CS where 21% of the patients unable to rise from a chair received rehabilitation, compared to 11% of the patients able to rise from a chair (p = 0.02).

**Table 1:** Characteristics of the included older medical patients.

	Total group				Group with mobility limitations at follow-up				Group with high mobilityat follow-up				*P*	
	N	missing	value		N	missing	value		N	missing	value			
Age, year, median (IQR)	369		77.9	(71.3; 84.5)	128		80.5	(73.9; 86.8)	188		76.3	(69.3; 83.0)		.0005
Female, number (%)	369		230	(62.3)	128		88	(68.8)	188		115	(61.2)		.17
Living with a partner, number (%)	366	(3)	122	(33.4)	128		26	(20.3)	186	(2)	83	(44.6)	<	.0001
Assisted living, number (%)	368	(1)	58	(15.8)	128		34	(26.6)	187	(1)	13	(7.0)	<	.0001
OMC, median (IQR)	359	(10)	22.0	(16.0; 26.0)	126	(2)	20.5	(15.0; 24.0)	184	(4)	24.0	(20.0; 26.0)	<	.0001
Weight at admission, kg median (SD)	331	(38)*	73.0	(61.2; 84.3)	109	(11)	71.5	(58.6; 84.9)	185	(3)	73.6	(63.7; 84.2)		.29
Education, primary school or less, number (%)	367	(2)	125	(34.1)	128		51	(39.8)	188		56	(29.8)		.08
Assistance with self-care activities, number (%)	357	(12)	96	(26.9)	125	(3)	60	(48.0)	183	(5)	20	(10.9)	<	.0001
Assistance with housekeeping, number (%)	356	(13)	234	(65.7)	123	(5)	109	(88.6)	183	(5)	90	(49.2)	<	.0001
Barthel Index 20, points median (IQR)	356	(13)	19.0	(17.0; 20.0)	120	(8)	17.0	(14.5; 19.0)	183	(5)	20.0	(19.0; 20.0)	<	.0001
NMS 14 days before admission. median (IQR)	368	(1)	7.0	(5.0; 9.0)	128		5.5	(3.0; 6.0)	187	(1)	9.0	(7.0; 9.0)	<	.0001
NMS at admission, median (IQR)	366	(3)	4.0	(2.0; 7.0)	128		3.0	(2.0; 5.0)	186	(2)	6.0	(3.0; 9.0)	<	.0001
EWS at admission, median (IQR)	369		4.0	(2.0; 6.0)	128		4.0	(2.0; 5.8)	188		4.0	(2.0; 5.8)		.58
History of fall within last year, number (%)	366	(3)	179	(48.9)	127	(1)	79	(62.2)	186	(2)	70	(37.6)	<	.0001
Use of walking aid at admission, number (%)	365	(4)	198	(54.1)	126	(2)	101	(80.2)	187	(1)	67	(35.8)	<	.0001
**Charlson co-morbidity Index:**	369		-		128		-		188		-		<	.0001
0, number (%)	-		130	(35.2)	-		36	(28.1)	-		81	(43.1)		-
1–2, number (%)	-		163	(44.2)	-		52	(40.6)	-		84	(44.7)		-
3+, number, (%)	-		58	(15.6)	-		40	(31.3)	-		23	(23.2)		-
**Reason for admission, number:**	369		-		-		-		-		-			-
Respiratory diseases, number (%)	-		105	(28.4)	-		-		-		-			-
Symptoms, or for observation^#^, number (%)	-		66	(17.9)	-		-		-		-			-
Cardiovascular diseases, number (%)	-		57	(15.4)	-		-		-		-			-
Endocrine diseases, number (%)	-		34	(9.2)	-		-		-		-			-
Genitourinary diseases, number (%)	-		30	(8.2)	-		-		-		-			-
Infectious diseases§, number (%)	-		22	(5.4)	-		-		-		-			-
Other}, number (%)	-		55	(15.4)	-		-		-		-			-
Length of stay, days median (IQR)	369		2.0	(1.0; 6.0)	128		4.0	(1.0; 8.0)	188		2.0	(1.0; 4.0)	<	.0001
HGS, woman, kg median (IQR)	229	(1)	17.4	(13.6; 21.1)	88		15.4	(11.1; 18.6)	115		19.1	(16.3; 23;1)	<	.0001
HGS, men, kg median (IQR)	139		31.0	(24.1; 38.5)	40		25.8	(19.6; 32.7)	73		35.4	(28.6; 41.9)		.0001
GS, m/s (median IQR)	317	(29)	0.7	(0.5; 0.9)	98		0.5	(0.4; 0.6)	183		0.8	(0.6; 1.0)	<	.0001
Patients without walking ability, number	23		-		16		-				-		<	.0001
Chair Stand	350	(19)	-		118	(10)	-		183	(5)	-			-
CS+, number (%)	228		-		-		38	(32.2)	-		168	(91.8)	<	.0001
CS-, number (%)	122		-		-		80	(67.8)	-		15	(8.2)	<	.0001
CAS, median (IQR)	366	(3)	6.0	(6.0; 6.0)	127	(1)	6.0	(4.0; 6.0)	187	(1)	6.0	(6.0; 6.0)	<	.0001

Admission characteristics of (left to right): all patients, patients with mobility limitations at follow-up, and patients with high mobility at follow-up, with p-values for mobility group-differences. OMC: The Short Orientation-Memory-Concentration Test, NMS: New Mobility Score, EWS: Early-Warning-Score, HGS: Handgrip strength, GS: gait speed CS+: patients able to rise with arms crossed in front of chest, CS-: patients unable to rise with arms crossed in front of chest, CAS: The Cumulated Ambulation Score *: only patients able to stand independent were weighed, #ICD-10 Chapter XVIII diagnosis (R00-R99) and Chapter XXI diagnosis (Z00-Z99) collapsed;§:ICD-10 Chapter I diagnosis (A00-B99),}: collapsed ICD-10 chapters with the fewest patients (4% or less of the total study sample each).

### Association between physical performance measures and limited mobility at follow-up

The associations between the performance measures at admission and limited mobility at follow-up are shown in Table 2. Unadjusted analyzes showed that the four performance measures at admission had strong and significant (p<0.001) associations with limited mobility at follow-up. The associations remained unchanged when adjusting for potential confounders (Table 2). The potential confounders had p-values >0.01 in the fully adjusted model, except for Charlson Comorbidity Index, which was significant with p-values ≤0.01 in models including CAS, GS, and CS. Trends were found for OMC in the models including HGS_M_ (p = 0.03), and CAS (p = 0.05); Charlson Comorbidity Index in the model including HGS_W_ p = 0.02, and age p = 0.02 in the model including CAS. When testing for modifiers, non-significant interactions were found except for CS where a borderline significant interaction was found with sex (p = 0.03). A backwards selection from a logistic regression model on all the four physical performance measures resulted in a model including only CS: OR = 0.13 (95%CI 0.06–0.28), (p< 0.0001) and GS: OR = 0.48 (95%CI 0.36–0.65) (p< 0.0001),

**Table 2:** Logistic regression models for mobility limitations in older medical patients 30 days after discharge.

Physical performance measure	N (Events*)	Model 1 OR (95% CI)	R^2^ Model 1	P**	N (Events*)	Model 2 OR (95% CI)	R^2^ Model 2	P**
HGS_M_, kg.	113 (40)	0.90 (0.86–0.95)	0.16	< .0001	111 (40)	0.91 (0.85–0.97)	0.25	< .0002
HGS_W_, kg.	203 (88)	0.86 (0.81–0.91)	0.15	< .0001	199 (86)	0.88 (0.83–0.94)	0.20	< .0001
GS, per 0.2 m/s	281 (98)	0.35 (0.26–0.46)	0.30	< .0001	275 (96)	0.32 (0.23–0.44)	0.35	< .0001
GS_NW_, per 0.2 m/s	297 (114)	0.33 (0.25–0.43)	0.36	< .0001	291 (112)	0.31 (0.22–0.42)	0.41	< .0001
CS	301 (118)	0.04 (0.02–0.08)	0.39	< .0001	295 (116)	0.04 (0.02–0.09)	0.45	< .0001
CAS, points	314 (127)	0.49 (0.38–0.64)	0.14	< .0001	308 (125)	0.54 (0.42–0.71)	0.22	< .0001

OR: odds ratio, 95%CI: 95% confidence interval. HGS_W_: handgrip strength, women, HGS_M_: handgrip strength, men, GS: gait speed, GS_NW_: inclusive patients without walking capacity at admission, assigned gait speed 0 m/s, CS: chair stand, ability to rise from a chair with arms crossed in front of chest, CAS: The Cumulated Ambulation Score,

* Events: patients with mobility limitations at follow-up (DEMMI < 62) (point), Model 1: univariate model, Model 2: adjusted for age, gender, The Short Orientation-Memory-Concentration Test, the Early-Warning- Score and the Charlson Comorbidity Index. Handgrip strength is stratified by gender. R^2^: R-squared for model 1 and 2, respectively.

** p-value for the association between each of the physical performance measures and the probability for mobility limitation at follow-up

Hosmer-Lemeshow tests were non-significant except for two cases the fully adjusted model including GS and CS, respectively. When investigating the goodness of fit of the GS model, a non-significant interaction between Charlson Comorbidity Index and sex was found, resulting in a non-significant goodness of fit and the estimate of GS (OR) did not change. For the CS model including the borderline significant interaction between sex and CS also resulted in a non-significant goodness of fit. Tests for linearity of HGS_M_, HGS_W_, GS and CAS were non-significant in all models.

The raw data showing the relationship between the total DEMMI score at follow-up and each of the physical performance scores at admission is shown in the S1 Fig.

### Prognostic accuracy

**Fig 2:**
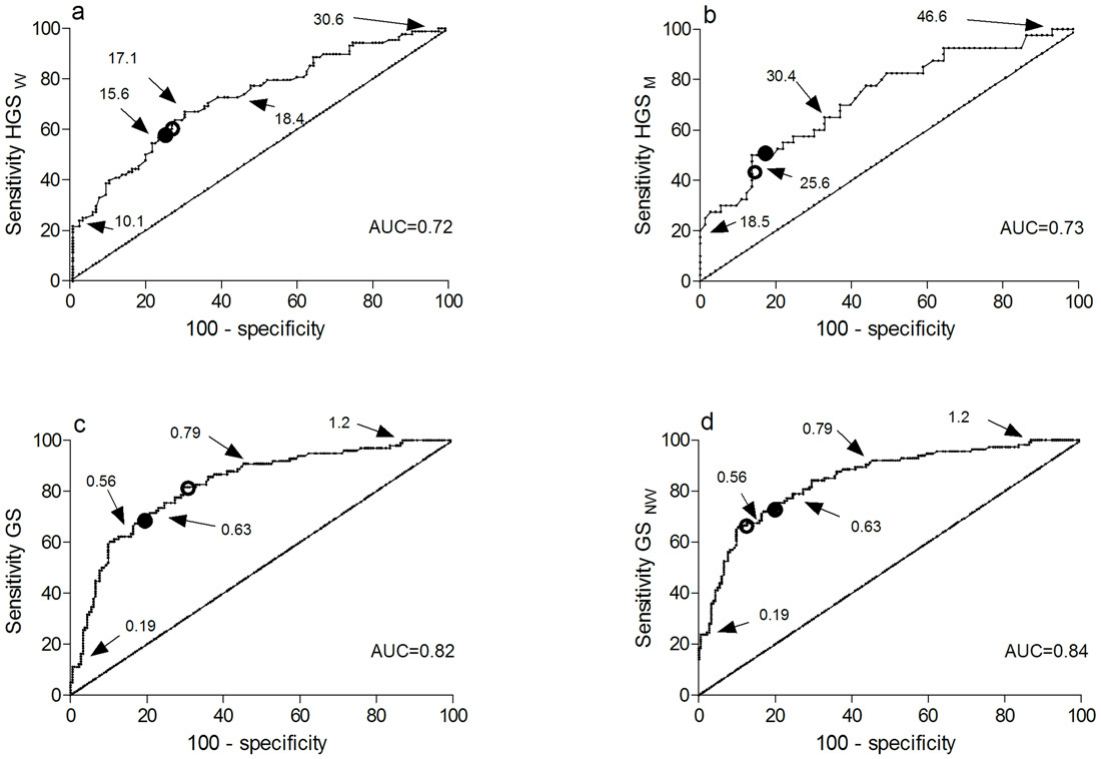
Receiver-operator characteristic (ROC) curves showing sensitivity and 100-specificity for prediction of mobility status 30 days after discharge, according to varying cut-offs for gait speed and handgrip strength. To illustrate the range of data and to show how the different cut-offs influence the sensitivity and specificity in the present study, selected absolute values are shown with arrows. Preselected cut-offs are marked with black circles, and cut-off based on Youden Index are shown with open circles. HGS_W_: handgrip strength (kg), women, HGS_M_: handgrip strength (kg), men, GS: gait speed (m/s), GS_NW:_ Including patients without walking ability at admission (m/s), AUC: area under the curve

ROC curves for HGS_w_, HGS_M_ and GS are shown in Fig 2a–2d. The area under the curves for HGS_w_, HGS_M_, and GS were above 0.7, with GS having the highest accuracy for discriminating mobility status at follow-up (GS: 0.82, (95%CI 0.77–0.88) HGS_W_: 0.72, (95%CI 0.65–0.79); HGS_M_: 0.73, (95%CI 0.63–0.83)). Missing scores in patients without walking capacity (GS_NW_) were replaced with the score 0 m/s, and the analysis was repeated. The inclusion of patients without walking capacity (n = 16) resulted in a slightly higher area under the curve: 0.85, (95%CI 0.80–0.89), as all patients had limited mobility at follow-up. Cut-offs based on the Youden Index for HGS_w_, HGS_M_, GS, and GS_NW_ were 16.70 kg, 25.03 kg, 0.69 m/s and 0.51 m/s respectively (Fig 2). Combining the four physical performance measures in a single algorithm (HGS_w_, HGS_M_, GS_NW_, CS, and CAS) yielded an area under the curve of 0.89, (95%CI 0.84–0.93), and a sensitivity of 81.31 and a specificity of 84.83 based on the Youden Index.

Table 3 shows the prognostic accuracy for the physical performance measures in relation to limited mobility at follow-up. The CS, GS, and GS_NW_ were able to identify patients with limited mobility at follow-up with sensitivity estimates between 67.8 and 72.8. The sensitivity of CAS, HGS_w_ and HGS_M_ was low (40.2 to 56.8). However, the specificity estimates of all physical performance measures were between 75.7 and 92.0.

**Table 3:** Prognostic accuracy for mobility limitations in older medical patients 30 days after discharge.

Physical performance measures	N (Events*)	AUC	Sensitivity (95% CI)	Specificity (95% CI)	PPV (95% CI)	NPV (95% CI)	LR+ (95% CI)	LR- (95% CI)
HGS_M_	113 (40)	0.73 (0.63–0.83)	50.0 (33.8–66.2)	80.8 (69.9–89.1)	58.8 (40.7–75.4)	74.7 (63.6–83.8)	2.6 (1.5–4.6)	0.6 (0.4–0.9)
HGS_W_	203 (88)	0.72 (0.65–0.79)	56.8 (45.8–67.3)	75.7 (66.8–83.2)	64.1 (52.4–74.7)	69.6 (60.7–77.5)	2.3 (1.6–3.4)	0.6 (0.4–0.7)
GS	281 (98)	0.82 (0.77–0.88)	68.4 (58.2–77.4)	81.4 (75.0–86.8)	66.3 (56.3–75.4)	82.8 (76.5–88.0)	3.7 (2.6–5.1)	0.4 (0.3–0.4)
GS_NW_	297 (114)	0.85 (0.80–0.89)	72.8 (64.6–80.7)	81.4 (75.0–86.8)	70.9 (61.8–79.0)	82.8 (76.5–88.0)	3.9 (2.8–5.4)	0.3 (0.2–0.5)
CS	301 (118)	-	67.8 (58.6–76.1)	91.8 (86.8–95.3)	84.2 (75.3–90.9)	81.6 (75.6–86.6)	8.3 (5.0–13.6)	0.4 (0.3–0.5)
CAS	314 (127)	-	40.2 (31.6–49.2)	92.0 (87.1–95.4)	77.3 (65.3–86.7)	69.4 (63.2–75.0)	5.0 (2.9–8.5)	0.7 (0.6–0.8)

* Events: patients with mobility limitations at follow-up (DEMMI< 62 points), AUC: area under the curve, 95% CI: 95% confidence interval, PPV: positive predictive value, NPV: negative predictive value, LR+: positive likelihood ratio, LR-: negative likelihood ratio, HGS_W_: handgrip strength, women, HGS_M_: handgrip strength, men, GS: gait speed, GS_NW_: inclusive patients without walking capacity at admission, assigned gait speed 0 m/s, CS: chair stand, CAS: The Cumulated Ambulation Score. Cut-off values for physical performance measures, which differentiate poor performance from high performance; HGS_W_: ≤16 kg, HGS_M_: ≤26 kg, GS: ≤0.6 m/s; CS: not able versus able to rise from a chair with arms folded in front of chest, CAS: < 6 points. HGS_W_, HGS_M_, and GS were based on cut-offs recommended in the literature.

### Sensitivity to missing data

The sensitivity of the results to patients with missing data in GS and CS was investigated. Missing data from patients unable to participate in the assessment due to physical problems related to the acute illness were replaced with scores below the cut-offs. The sensitivity was slightly improved for both measures; (GS: 75.2, (95%CI 67.6–82.8) and CS: 68.9, (95%CI 60.1–77.1)). The specificity was unchanged for CS; (90.8 (95%CI 86.7–95.0)) and slightly reduced for GS; (GS: 79.3 (95%CI 73.5–84.8)).

Finally, to evaluate the sensitivity of our results to patients who did not participate in the follow-up visit, the univariate analyzes and the calculation of sensitivity and specificity were repeated. Patients with missing DEMMI caused by deterioration in health or death at follow-up were analyzed as limited mobility, and missing DEMMI caused by patients who declined or were lost to follow-up were analyzed as either limited mobility or high mobility. This did not change our results substantially, as the estimates from the sensitivity analyzes were all within the confidence intervals from the primary analyzes.

## Discussion

The present study has two primary findings. First, each of the physical performance measures was strongly associated with the mobility level 30 days after discharge. These associations were all linear on the logit scale, and were not modified by: age, gender, cognitive status, and the Charlson Comorbidity Index. Second, the ability to identify patients with limited mobility 30 days after discharge was highest for CS and GS with lower prognostic accuracy for the HGS_M_, HGS_W_ and CAS, at the prespecified cut-offs.

Regardless of the acute medical illness leading to hospitalization our results were similar to those reported in both cross sectional and prospective studies in community-living older adults [9–13,31], showing independent associations between the level of different physical performance measures and self-reported mobility and ADL limitations. Despite heterogeneity in study designs, physical performance measures and outcome measures, the studies showed that physical performance relates to current and future functional disability in older people. As such, physical performance measures have the potential to be used systematically in identification of older adults at risk of developing dependency. However, few studies have evaluated the predictive ability of physical performance measures in older medical patients with diverse ranges of medical conditions and functional levels [6,8,32–34], and most of these studies have investigated the predictive ability related to ADL dependence [6,7,32–34], and not mobility limitations [8].

In this study, mobility limitations were quantified with an objective measure developed specifically for older medical patients and with established normative values from community-living older adults [41]. The main literature within this field used self-reported measures of mobility [8–10,12,13], which may be associated with a risk of recall bias. The DEMMI is valid, reliable, and responsive across health-care settings, it is free from floor and ceiling effects, and it is not affected by recall bias, which supports the validity of the mobility status at the follow-up [36–40]. Finally, the DEMMI was assessed in the patients’ home thereby reflecting mobility restrictions in their every-day environment.

Measurements that provide continuous data without a cut-off value have limited feasibility in the clinical setting. Accordingly, cut-offs for the HGS and GS that are recommended for screening of older people at risk for mobility limitation and adverse health outcomes were used. As shown in Fig 2, choosing the cut-offs for HGS_M_, HGS_W_, GS, and GS_NW_ that maximized sensitivity and specificity resulted in minor changes in the prognostic accuracy estimates. Our findings support the cut-offs for HGS_M_ and HGS_W_ reported in the literature and emphasize that the cut-off for GS is lower in frail and hospitalized older populations than in healthy community-living populations where a cut-off of 0.8 m/s is frequently used. The cut-off that maximized sensitivity and specificity for GS differed slightly from the a-priori based cut-of (0.6 m/s) depending upon the inclusion or exclusion of patients who were not able to walk at admission. Importantly, no standard exists as to what constitutes an acceptable level of prognostic accuracy, as it depends on the specific clinical situation and the consequences related to misclassification of patients [57]. In the acute hospital setting, misclassification ultimately implies that “older at-risk patients” are not offered further evaluation and rehabilitation and vice versa. With the growing elderly population and the associated health-care costs, there is a need for measures that have a high probability of correctly identifying older at-risk patients to direct care and treatment to those with the greatest needs. This implies that sensitivity, specificity, positive predictive value and negative predictive value all need to be at an acceptable level to minimize misclassification. In our study, CS and GS showed the highest predictive accuracy of the measures no matter how the cut-offs were selected. Furthermore, the algorithm based on all the physical performance measures showed a lower specificity but an increased sensitivity compared to the algorithm based on CS or GS_NW_ solely. However, the small clinical gain from administering an algorithm based on four different performance measures compared to one measure only would likely be outweighed by the increased time demand. Accordingly, we believe that a simple and fast measure that supports the patient flow in the ED is preferable [58]

The median gait speed was low in our patients, which is consistent with findings from studies performed in similar populations [2,26,27,32]. Accordingly, the choice of using a low cut-off recommended for frail older populations seems reasonable [10,11,26,27,59]. Comparable to our feasibility study [43] around 14% of the included patients had missing GS scores mainly due to physical problems caused by the medical condition and permanent use of a wheelchair. Other studies have reported that 36–49% of older medical patients were unable to perform the GS at admission to acute care wards [2,26], questioning the use of GS as an early marker for mobility limitation. Differences in the eligibility criteria may explain the higher feasibility in our study as we in contrast to the study of de Buyser et al. [2] excluded patients with cancer, terminal illness and patients transferred to Intensive Care Units. Nevertheless, we believe that a risk evaluation in many of the excluded patients will not contribute to further information, as these patients are obvious at high risk of adverse health events. The prognostic accuracy of GS was acceptable and maintained after accounting for patients without walking capacity and severe illness. This confirms the applicability of GS in the acute setting as a simple measure of mobility limitations after discharge.

The CS was superior to GS in predicting mobility limitations at follow-up. The assessment of CS is fast and applicable in patients without walking capacity and does not require special equipment, making CS even easier to implement in the clinical setting. Noteworthy, in older community-living adults, inability to rise from a chair was associated with the highest mortality risk [14]. In contrast to prior research in older medical patients [6–8], the predictive ability of the measures reported in our study was based on assessments at admission because early identification of at-risk patients followed by a relevant intervention may have a major impact on functional decline during hospitalization and failure to recover post discharge. Furthermore, up to 50% of older medical patients are discharged the day after their admission [35]. Referral of these patients to post discharge rehabilitation, therefore, depends on early identification of potential rehabilitation needs.

Our results showed that poor performance on measures that involve the lower extremity (GS, CS and CAS) in contrast to HGS had a higher prognostic accuracy (LR+ > 3.6) for mobility limitations at follow-up in older medical patients. As the DEMMI comprises tasks that primarily involve the lower extremities, including, chair rise, bed transfer and walking capacity, this finding was expected [36]. In addition, and similar to our findings, Onder et al. showed that lower extremity measures, particularly GS showed greater predictive ability than HGS for future self-reported mobility disability in disabled older community-living women [31]. Nevertheless, HGS is proposed to be a surrogate measure of physical reserve capacity [60], sarcopenia [15,18] and mobility limitations [15,18,46], and associations between low HGS and various adverse health outcomes have indeed been reported [14,29,34]. Studies evaluating the application of HGS cut-offs in predicting increased dependence in older acutely admitted medical patients are few and results not convincing. For example, García-Peña et al. found that assessment of HGS at admission to an acute care ward predicted a decline in the Barthel Index with a sensitivity of 56% and a specificity of 91% in elderly males, but not in females [34].

Finally, the CAS showed an acceptable positive likelihood ratio in the present study, indicating that a patient with a poor test result is 5 times more likely to have mobility limitations at follow-up. However, the sensitivity of the CAS was low as CAS showed a ceiling effect in this population, making the CAS inappropriate as an early marker for mobility limitations at follow-up.

### Strengths and limitations

The major strengths of this study are: the large study sample and the low attrition; the objective measure of mobility; the quantification of mobility limitations based on normative values for older community-living adults; the use of literature-based cut-offs, which adds important knowledge of their relevance and predictive ability in a hospitalized population. A limitation is the high rate of patients who declined to participate and who were more dependent in walking both 14 days prior to and on the day of admission to the hospital. This is a well-known challenge of conducting research in hospitalized populations, and it is difficult to avoid but it indicates that we may have a selected population (selection bias), which could influence the generalizability of our result. Moreover, the patients with missing data at baseline and the patients who did not participate in the follow-up because of refusal, deterioration in health, and death before the follow-up could bias our results. However, the sensitivity analyzes did not change the results. The generalizability of the study results might be limited due to our eligibility criteria, which were focused on the patients´ ability to give informed content, the logistics in completing the measurements before discharge, and to screen out patients with severe illness. However, we believe that physical performance measures are applicable in many of the excluded patients and that the association between the measures from admission to follow-up might be similar. Finally, our results need to be replicated in a dedicated validation-sample of older medical patients.

## Conclusion

In older patients admitted for acute medical illness, measurements of handgrip strength, gait speed, modified chair stand test and the Cumulated Ambulation Score at admission to an emergency department were strongly associated with mobility limitations 30 days after discharge. These associations were not modified by any of the covariates: age, gender, cognitive status, the severity of the acute medical illness, and the Charlson Comorbidity Index supporting the independent value of poor physical performance when evaluating the risk for mobility limitations 30 days after discharge. Assessments of chair stand and gait speed had the highest prognostic accuracy for limited mobility 30 days after discharge with lower prognostic accuracy of handgrip strength and the Cumulated Ambulation Score. Hence, simple measures of physical performance, particularly chair stand and gait speed have the potential to assist health-care professionals in allocating health care resources to older patients at risk of losing independence.

## Supporting Information

S1 Fig Raw data showing the relationship between DEMMI 30 days after discharge and the physical performance measures at admission. The horizontal line displays the cut-off for mobility limitations (DEMMI score < 62). The vertical lines display the different cut-offs from the literature for HGS_W_: (16 kg, 20 kg, 21 kg), HGS_M_: (26 kg, 30 kg, 37 kg), GS: (0.6 m/s, 0.8 m/s, 1.0 m/s), which have been reported in earlier studies of community-living older adults to predict mobility limitations and adverse health events. The figures illustrate the consequences of using higher cut-offs for HGS_W_, HGS_M_, and GS in this population, by showing the number of patients (n), who would be classified as “poor performers” (risk-patients) at admission by using a higher cut-off. The numbers in brackets show patients with missing items in DEMMI, who were included in the analysis, as the missing value did not affect the mobility status (mobility limitations versus high mobility) at follow-up. DEMMI: de Morton Mobility Index, HGS_W_: handgrip strength, women, HGS_M_: handgrip strength, men, GS: gait speed, CS+: able to rise from a chair with arms folded in front of chest, CS-: unable to rise from a chair with arms folded across the chest, CAS: The Cumulated Ambulation Score.
(TIF)

S1 Raw Data Raw data for the study. (XLSX)
