# Supplementary figures and images for: Prediction of Mobility Limitations after Hospitalization in Older Medical Patients by Simple Measures of Physical Performance Obtained at Admission to the Emergency Department
Source: PLoS One. 2016 May 19;11(5):e0154350. doi: 10.1371/journal.pone.0154350 (PMC4873238; doi:10.1371/journal.pone.0154350)

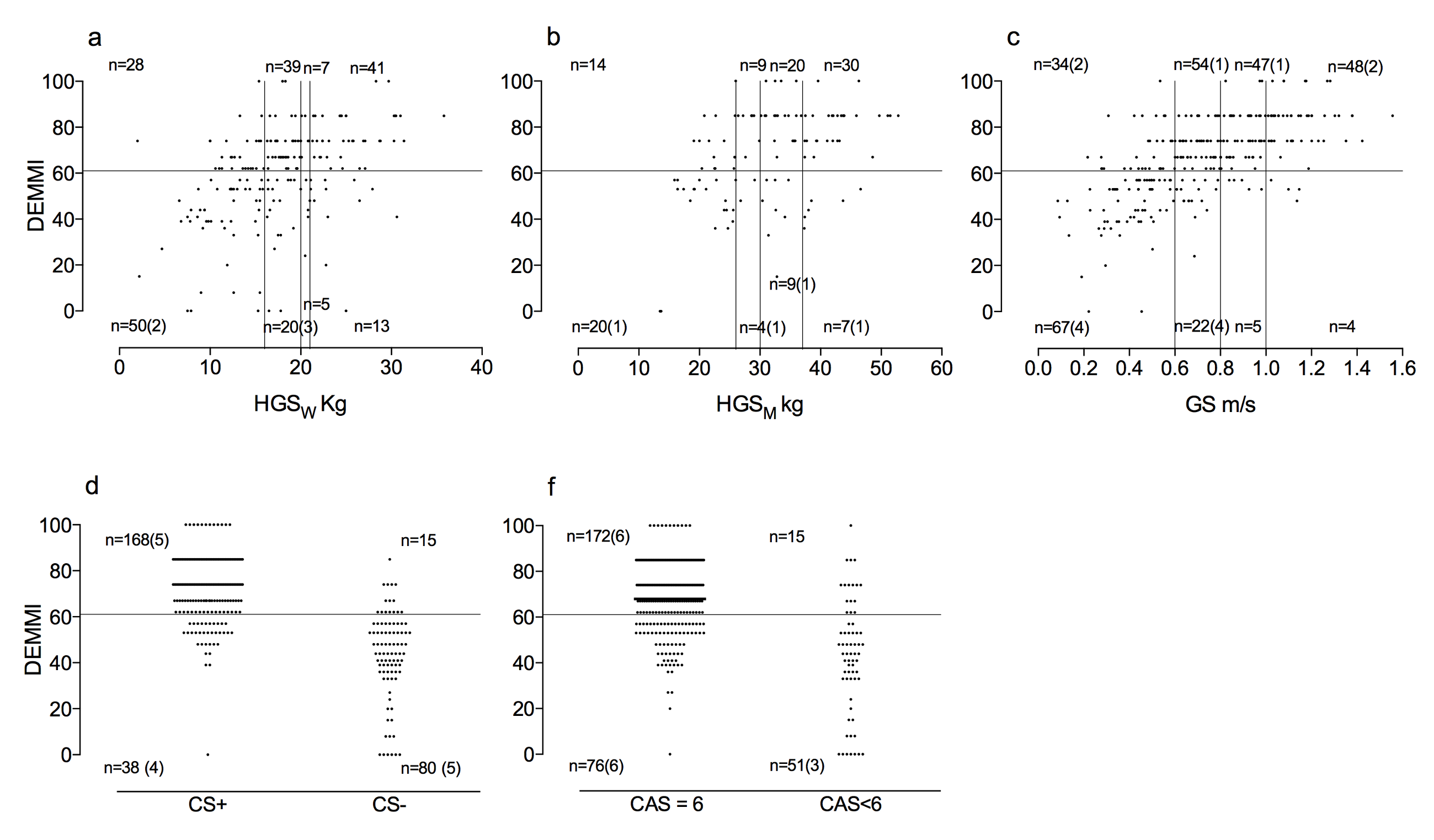

Supplement: S1 Fig — The horizontal line displays the cut-off for mobility limitations (DEMMI score < 62). The vertical lines display the different cut-offs from the literature for HGSW: (16 kg, 20 kg, 21 kg), HGSM: (26 kg, 30 kg, 37 kg), GS: (0.6 m/s, 0.8 m/s, 1.0 m/s), which have been reported in earlier studies of community-living older adults to predict mobility limitations and adverse health events. The figures illustrate the consequences of using higher cut-offs for HGSW, HGSM, and GS in this population, by showing the number of patients (n), who would be classified as “poor performers” (risk-patients) at admission by using a higher cut-off. The numbers in brackets show patients with missing items in DEMMI, who were included in the analysis, as the missing value did not affect the mobility status (mobility limitations versus high mobility) at follow-up. DEMMI: de Morton Mobility Index, HGSW: handgrip strength, women, HGSM: handgrip strength, men, GS: gait speed, CS+: able to rise from a chair with arms folded in front of chest, CS-: unable to rise from a chair with arms folded across the chest, CAS: The Cumulated Ambulation Score. (TIF) [file pone.0154350.s001.tif]
